# A Facile Approach for the Synthesis of Zn_2_SnO_4_/BiOBr Hybrid Nanocomposites with Improved Visible-Light Photocatalytic Performance

**DOI:** 10.3390/nano8050313

**Published:** 2018-05-09

**Authors:** Tiekun Jia, Ming Liu, Dongsheng Yu, Fei Long, Shuyi Mo, Zhao Deng, Weimin Wang

**Affiliations:** 1 School of Materials Science and Engineering, Luoyang Institute of Science and Technology, Luoyang 471023, China; liumingming4455@163.com (M.L.); dongsh_yu@163.com (D.Y.); 2 Guangxi Ministry-Province Jointly-Constructed Cultivation Base for State Key Laboratory of Processing for Non-ferrous Metal and Featured Materials, Guilin University of Technology, Guilin, 541004, China; longf@glut.edu.cn (F.L.); moshuyi@glut.edu.cn (S.M.); 3 State Key Lab of Materials Synthesis and Processing, Wuhan University of Technology, Wuhan 430070, China; wangwm@hotmail.com

**Keywords:** nanocomposites, Zn_2_SnO_4_/BiOBr, visible light, photocatalytic performance

## Abstract

In this study, a novel Zn_2_SnO_4_/BiOBr hybrid photocatalyst was prepared via a mild hydrothermal synthesis combined with a chemical deposition method. The morphological structure, chemical composition, crystal structure, and optical properties were comprehensively characterized by a series of measurement techniques. Morphological observation showed that fine Zn_2_SnO_4_ nanoparticles were anchored on the nanoplate surface of a flower-like BiOBr 3D hierarchical structure. The experimental results of UV-vis diffuse reflection spectroscopy revealed that the visible-light absorptive capacity of the Zn_2_SnO_4_/BiOBr hybrid photocatalyst was promoted, as compared to that of pure Zn_2_SnO_4_. Evidenced by electro-negativity theoretical calculation, Zn_2_SnO_4_ and BiOBr possessed matched band edges for accelerating photogenerated charge separation at the interface. The Zn_2_SnO_4_/BiOBr hybrid photocatalyst exhibited enhanced photocatalytic performance in the degradation of Rhodamine B (RhB) under visible light irradiation. According to the band energy structure and the experimental results, the enhanced photocatalytic performance was ascribed to the improved visible-light absorptive capacity and the contact interface between Zn_2_SnO_4_ nanoparticles and BiOBr nanoplates, being able to favor the prompt charge migration and suppress the recombination of photogenerated carriers in the hybrid system.

## 1. Introduction

Nowadays, photocatalysis is considered to be an effective and sustainable approach for resolving the disturbing issue of the energy crisis and environmental pollution. Owing to the high electrical conductivity and electron mobility [1,2,3,4], Zn_2_SnO_4_ (ZTO) has been confirmed the diversity in the photodegradation of organic contaminants [5,6,7,8,9,10], the evolution of hydrogen from ethanol solution [11], and the reduction of CO_2_ to CH_4_ [12]. For instance, Lou et al. demonstrated the photodegradation of three water-soluble organic dyes, using ZTO nanocrystals as photocatalyst under UV irradiation [5]. Moreover, the photodegradation of rhodamine B (RhB) [6], methyl blue [7,8], methyl orange [9], and direct black 38 azo-dye [10] was successfully achieved over ZTO nanostructured photocatalysts under UV irradiation. Besides these examples, as reported in the previous literature [11], the photocatalytic H_2_ production from methanol solution was successfully accomplished using a ZTO nanostructured material as a photocatalyst under UV irradiation. Despite of the merits mentioned above, ZTO has a wide bandgap (~3.6 eV), leading to the fact that it can only absorb a small portion of the total irradiated natural sunlight. Thus, most of the above mentioned studies on ZTO nanostructured material addressed the enhancement of photocatalytic activity under UV light illumination, owing to its wide band gap. That is to say, the spectral response of bare ZTO nanostructured photocatalysts is still limited to UV region, and the utilization efficiency of solar energy is not desirable for the low portion of UV light in the total solar energy. From another point of view, like other single-component photocatalysts, ZTO also suffers from the fast recombination rate of the photo-generated charge carriers, which is considered to be another critical cause for hindering bare ZTO photocatalysts from acquiring excellent photoactivity. Based on the aforementioned research background, it is particularly essential to design a simple and efficient strategy for achieving visible-light-active ZTO based photocatalysts with high photocatalytic efficiency.

Coupling semiconductor photocatalysts with matched band energy levels has been proven to be an effective strategy for facilitating efficient photogenerated charge separation [12,13,14,15,16,17,18,19,20,21,22]. Owing to its desirable band gap (2.7 eV) and peculiar layered structure constituted by interlacing [Bi_2_O_2_] slabs with double bromine slabs, BiOBr is emerging as a relatively efficient and stable visible-light-activated photocatalyst. As a sensitizer, BiOBr can extend the visible light response range of wide band gap semiconductors, and separate the photogenerated carriers efficiently. Until now, a series of coupled photocatalysts containing BiOBr, such as, ZnO/BiOBr [18], Ni_2_FeO_4_/BiOBr [19], BiPO_4_/BiOBr [20], TiO_2_/BiOBr [21], ZnWO_4_/BiOBr [22] have been reported to show improved photocatalytic performance for degrading organic pollutants. According to previous reports, BiOBr has matched the energy levels of the valence band (VB) and conduction band (CB) with those of ZTO, which provides a theoretical basis for the migration of photoinduced charge carriers in the photocatalytic process. Considering the above mentioned characteristics, ZTO/BiOBr hybrid structures are anticipated to possess high photocatalytic efficiency and photostability compared with pure ZTO. To the best of our knowledge, the preparation and investigation on ZTO/BiOBr hybrid structure has not been reported.

Herein, we developed a facile approach to fabricate ZTO/BiOBr nanocomposites via a hydrothermal synthesis followed by in situ chemical deposition method. According to the determined experimental procedure, a series of ZTO/BiOBr hybrid nanocomposites was obtained by loading different amounts of BiOBr nanoplates. Compared with pure ZTO, the resulting ZTO/BiOBr nanocomposites exhibited wider visible light response range and higher photocatalytic performance toward the degradation of RhB solution. The proposed mechanism of the enhanced photocatalytic performance was discussed based on the experimental results and energy band structure analysis.

## 2. Results and Discussion

**Figure 1:**
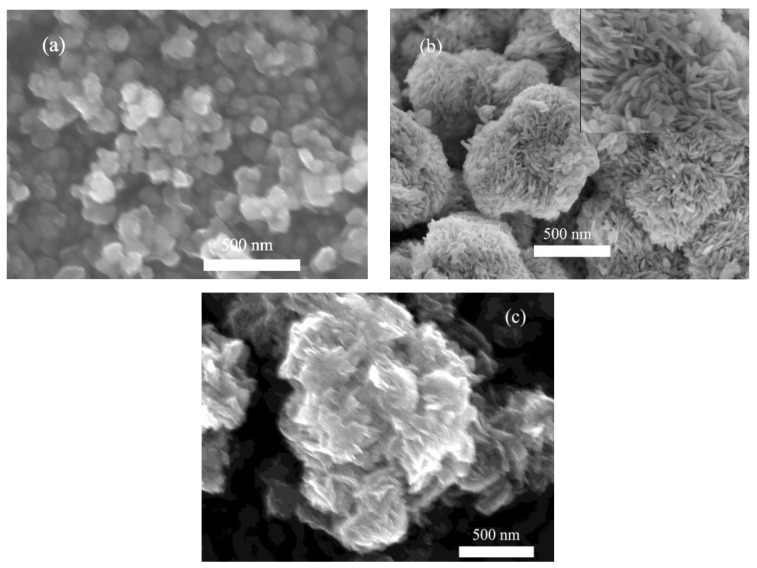
FESEM images of (**a**) pure ZTO; (**b**) pure BiOBr; and (**c**) 1ZTO/1BiOBr.

The morphology and microstructure of pure ZTO, pure BiOBr, and 1ZTO/1BiOBr samples were investigated by SEM and TEM images. As displayed in Figure 1a, pure ZTO consisted of abundant fine nanoparticles with sizes ranging from 25–40 nm. From Figure 1b, it is clearly observed that pure BiOBr exhibited flower-like 3D hierarchical structure assembled by substantive nanoplates with thicknesses of 5–8 nm. As for the 1ZTO/1BiOBr sample (Figure 1c), it can be distinctly observed that fine ZTO nanoparticles stacked onto the nanoplate surface of flower-like BiOBr 3D hierarchical structure. Comparatively, the morphology of BiOBr was substantially modified after the introduction of ZTO nanoparticles; the size of nanoplates decreased also. Notably, the linked and intercrossed nanoplates endowed the 1ZTO/1BiOBr sample with an exceptive structure, which resulted in the formation of nano- and macro-pores. The existence of pores and the interface between ZTO and BiOBr are favorable for visible light harvesting and the transfer of photoinduced electron-hole pairs.

**Figure 2:**
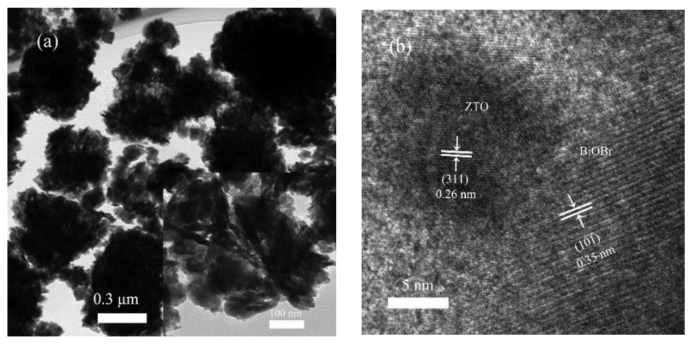

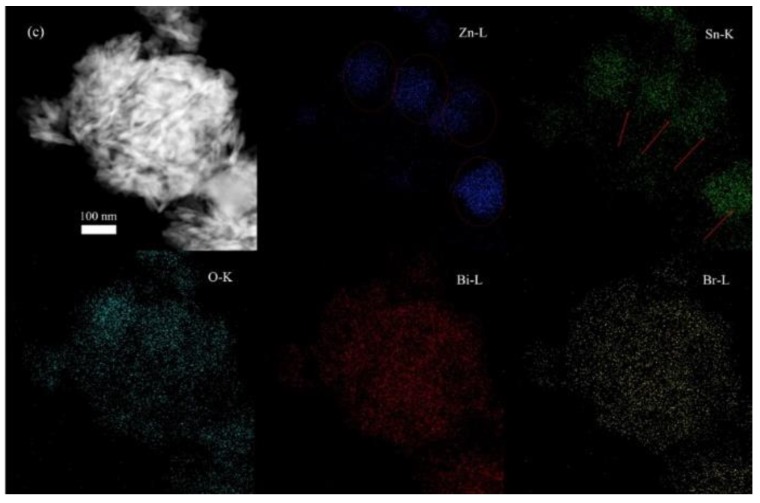
(**a**) TEM image and (**b**) HRTEM image of 1ZTO/1BiOBr; and (**c**) the corresponding EDS mapping of Zn, Sn, O, Bi and Br elements.

Figure 2a presents a typical TEM image of the 1ZTO/1BiOBr sample, having 3D flower-like hierarchical structure with ZTO nanoparticles growing on the surface of BiOBr nanoplate. Clear and ordered lattice fringes with an interplanar distance of 0.35 nm are indexed to be (101) planes of BiOBr from Figure 2b, indicating high crystallinity. Another lattice fringe with d value of 0.26 nm belongs to (311) plane of ZTO. The above microstructural observation demonstrated that BiOBr nanoplates accreted onto the surface of ZTO nanoparticles, and the intimate contact at the heterojunction, was accomplished along the interface of ZTO nanoparticles and BiOBr nanoplates. Figure 2c shows the typical high angle annular dark field (HAADF)-scanning transmission electron microscope (STEM) image of the 1ZTO/1BiOBr sample. The result of elemental analysis validated the existence of Zn, Sn, O, Bi and Br, revealing a uniform distribution of the above elements throughout the hybrid system as well. Additionally, according to the Brunauer-Emmett-Teller (BET) method, the specific surface area of the ZTO and 1ZTO/1BiOBr samples can be estimated to be 69.5 m^2^/g and 46.4 m^2^/g respectively, indicating that the BET surface area of the 1ZTO/1BiOBr sample exhibits a slight decrease compared with that of pure ZTO photocatalyst.

**Figure 3:**
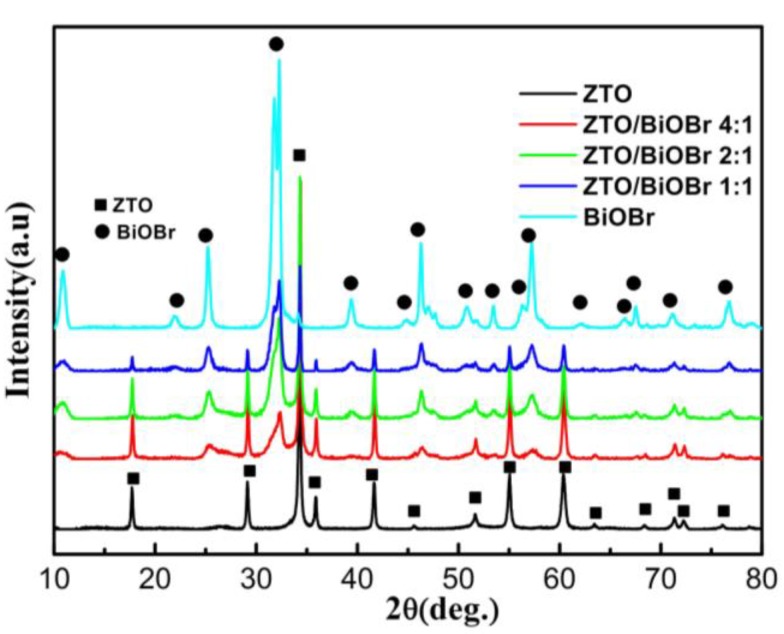
XRD patterns of ZTO, BiOBr, and ZTO/BiOBr nanocomposites.

Figure 3 presents the XRD patterns of ZTO, BiOBr and ZTO/BiOBr nanocomposites with different mass ratios. All the characteristic diffraction peaks of bare ZTO can be identified to the cubic Zn_2_SnO_4_ (JCPDS 74-2184). Four intense diffraction peaks at 2θ angles of 17.6°, 28.9°, 34.1°, 41.4° and 60.1° are in high accordance with (111), (220), (311), (400) and (440) planes of ZTO, respectively. For bare BiOBr sample, six characteristic diffraction peaks correspond respectively to the planes of (001), (011), (012), (110), (020) and (212), which agree well with the standard card of the tetragonal phase of BiOBr (JCPDS 09-0393). Two sets of diffraction peaks resulting from ZTO and BiOBr are observable in the ZTO/BiOBr spectra, indicating the coexistence of both ZTO and BiOBr phases in the nanocomposites. Moreover, the intensities of BiOBr tend to be greater with the decrease of ZTO. The grain size of the BiOBr and ZTO crystallites was calculated by means of the Debye-scherrer formula from the broadening of the (101) and (311) XRD reflections at 2θ of 25.304° and 34.319° respectively, after Kα_2_ correction. Full width at half-maximum (FWHM) values was obtained using the Highscore software program as 0.56° and 0.199°. The calculated particle sizes for the BiOBr and Zn_2_SnO_4_ crystallites are 14.4 nm and 41.3 nm respectively. The XRD result revealed that the obtained samples are of high crystallinty, agreeing well with that of TEM and HRTEM analysis. Additionally, no other diffraction peaks from other impurities are found in the ZTO/BiOBr nanocomposites, suggesting that the as-prepared samples are rather pure.

**Figure 4:**
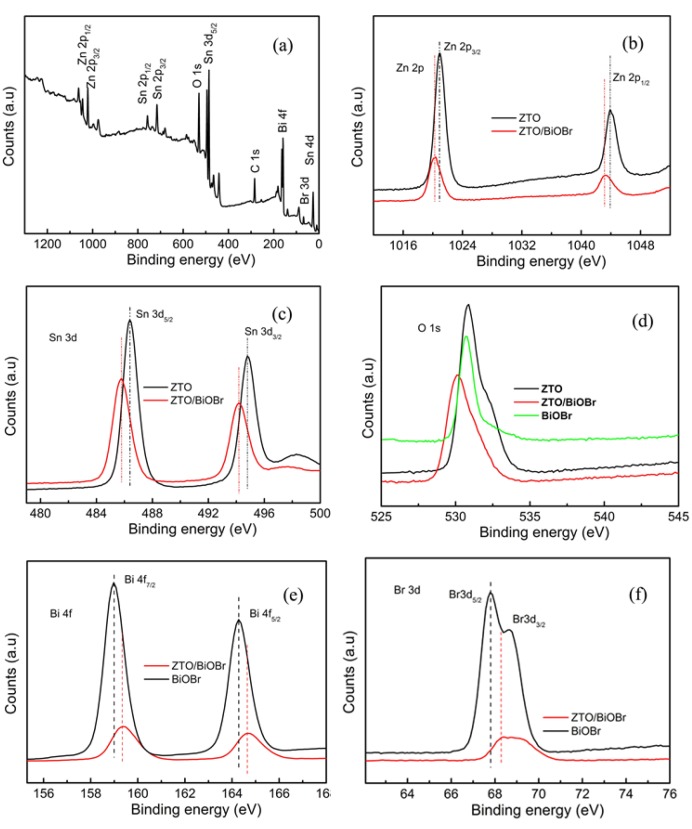
XPS spectra of pure ZTO, BiOBr, and 1ZTO/1BiOBr; (**a**) Survey spectrum; (**b**) Zn 2p; (**c**) Sn 3d; (**d**) O 1s; (**e**) Bi 4f; (**f**) Br 3d.

Figure 4a shows the XPS survey spectrum of the 1ZTO/1BiOBr sample. From Figure 4a, we can observe that the as-obtained 1ZTO/1BiOBr sample is composed of Sn, Zn, O, Bi and Br. High resolution XPS spectra of Zn 2p, Sn 3d, Bi 4f, Br 3d and O 1s are presented in Figure 4b–f. From Figure 4b, two typical peaks located at 1020.5 and 1043.4 eV for bare ZTO sample correspond to Zn 2p_3/2_ and Zn 2p_1/2_ respectively. When BiOBr nanoflakes were introduced into the composite system to form the heterojunction, the Zn 2p_1/2_ and Zn 2p_3/2_ peaks shifted slightly toward higher binding energies by ca. 0.2 eV. Double peaks located at 485.9 and 494.3 eV for bare ZTO sample (Figure 4c) are respectively attributed to Sn 3d_5/2_ and Sn 3d_3/2_. Similarly, the Sn 3d_5/2_ and Sn 3d_3/2_ peaks of the 1ZTO/1BiOBr sample exhibited an obvious shift toward high binding energies by ca. 0.5 eV in comparison with those of bare ZTO. From Figure 4d, we can find that the peak position of O 1s spectra for the 1ZTO/1BiOBr sample is different from the counterparts of bare ZTO and BiOBr, which is probably due to the presence of hybrid bonds of Bi-O, Zn-O and Sn-O in the nanocomposites. In terms of Bi 4f (158.9 eV for Bi 4f_7/2_ and 164.3 eV for Bi 4f_5/2_) and Br 3d (68.1 eV for Br 3d_5/2_ and 68.8 eV for Br 3d_3/2_) for the 1ZTO/1BiOBr sample (Figure 4e,f), the binding energies are a bit lower than the corresponding counterparts for pure ZTO and BiOBr. The phenomenon of the as-mentioned binding energy shift is most likely a result of the strong interaction between ZTO NPs and BiOBr nanoplates. From a theoretical point of view, the reinforcement of binding energies implies a weakened electron screening effect caused by decreased electron concentration, whereas the increase in electron concentration results in a decrease of binding energies, owing to the improved electron screening effect [23,24,25,26]. Based on the above demonstration, we think that the higher and lower shifts are respectively attributed to the decreased electron concentration of ZTO NPs and increased electron concentration of BiOBr nanoplates, due to the strong interaction between the ZTO NPs and BiOBr nanoplates, assisted by the interfacial charge carrier migration.

**Figure 5:**
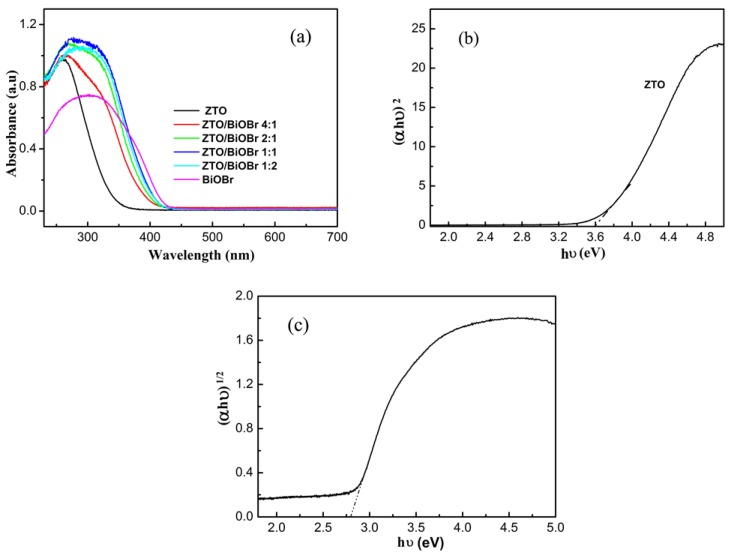
(**a**) UV-vis absorbance spectra of pure ZTO, BiOBr, and ZTO/BiOBr nanocomposites; (**b**) the derived plot of *(*αhν*)*^2^ versus hν from the absorption spectrum for pure ZTO; (**c**) the derived plot of (αhν*)*^1/2^ versus hν from the absorption spectrum for pure BiOBr.

In order to characterize the optical absorption properties, the obtained diffuse reflection spectra (DRS) are presented in Figure 5. The absorption edge was approximately located at the wavelength of 350 nm for pure ZTO photocatalyst, as displayed in Figure 5a. In addition, a steep slope was found for the absorption curve of pure ZTO, indicating the feature of strong UV light absorption capacity. Comparably, BiOBr exhibited visible light absorption with wavelengths of below 450 nm. After hybridization of ZTO nanoparticles with BiOBr nanoplates, an evident redshift was seen from the absorption edge of ZTO/BiOBr nanocomposites, as compared with pure ZTO, suggesting that BiOBr has the functionality of a visible light sensitizer. Therefore, we can infer that more photon efficiency will be acquired in a ZTO/BiOBr system, resulting in the generation of more photoinduced carriers for enhancing photocatalytic performance. Moreover, ZTO/BiOBr hybrid nanocomposites possessed enhanced UV light absorption, indicating the result of an absorbance superposition of the ZTO and BiOBr components.

The band gap energy can be determined according to the empirical equation, as follows: (αhν)^2/n^ = A(hν − E_g_) [27]. In the empirical equation, α, hν, E_g_ and A correspond to the absorption coefficient, photon energy, band-gap energy, and a constant. The n values are equal to 1 for ZTO (direct semiconductor) and 4 for BiOBr (indirect semiconductor), respectively, owing to their intrinsic characteristic of electronic transition. After calculation, the E_g_ values of pure ZTO and BiOBr were respectively estimated to be 3.58 eV and 2.79 eV from the corresponding plots of hν against (αhν)^2^ and hν against (αhν)^1/2^ (Figure 5b,c). Moreover, the band edge positions of ZTO and BiOBr can be determined through the empirical formulas [28] as follows:

E_CB_ = X − E_e_ − 0.5E_g_

E_VB_ = E_CB_ + E_g_

E_CB_ and E_VB_ respectively represent the conduction band (CB) and valence band potential. X belongs to the electronegativity of ZTO and BiOBr, whose values are 7.0 and 6.18 eV respectively. E_g_ and E_e_ refer to the band gap energy and energy of free electrons on the hydrogen scale (~4.5 eV) respectively. After calculation, the E_CB_ values of ZTO and BiOBr were determined to be 0.71 and 0.28 eV, while the E_VB_ values of ZTO and BiOBr were respectively determined to be 4.3 and 3.07 eV.

**Figure 6:**
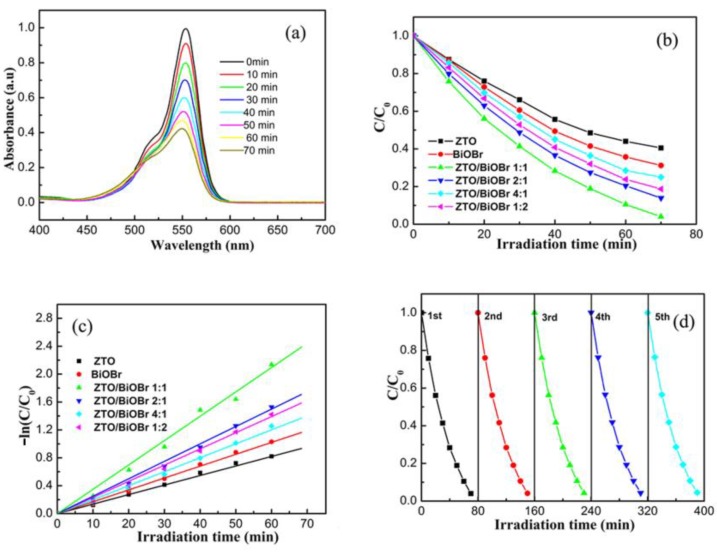
(**a**) Time dependent absorption spectra of RhB aqueous solution over pure ZTO; (**b**) the photodegradation performance of RhB solution over photocatalysts of pure ZTO, BiOBr, and ZTO/BiOBr nanocomposites; (**c**) the plots of −ln(C/C_0_) versus irradiation time for RhB degradation over pure ZTO, BiOBr, and ZTO/BiOBr nanocomposites; (**d**) cycling performance of the photodegradation of RhB solution over 1ZTO/1BiOBr.

Figure 6a shows the plots of absorbance with respect to irradiation time in the presence of pure ZTO. A stepwise decrease was found from the intensity of the absorption band at 554 nm, indicating that RhB solution was gradually photodegraded over ZTO photocatalyst. Additionally, a slight hypsochromic shift deviating from characteristic absorption band of RhB was also found, with prolonged illumination time during the photodegradation process, agreeing well with previous report [29]. Figure 6b displays the photodegradation activities of RhB over different photocatalysts. As seen in Figure 6b, pure ZTO and BiOBr exhibited low photocatalyitic performance, with only about 60% and 69% of the RhB photodegradation rate after 70 min of visible light illumination. After hybridizing ZTO with BiOBr, the photocatalytic performance of ZTO/BiOBr nanocomposites was obviously improved, as compared with that of single component photocatalyst. Specifically, the photodegradation rate of RhB continuously increased with an increase in BiOBr component, until the mass ratio of BiOBr reached up to 50%. The photodegradation rate of RhB over ZTO/BiOBr nanocomposites decreased steeply, relative to the maximum value (~96%), when the mass ratio of BiOBr exceeded the critical value of 50%. This phenomenon may be attributed to the cooperative roles between ZTO nanoparticles and BiOBr nanoplates exerting a remarkable influence on the charge carrier migration between two semiconductors. At a low mass ratio of BiOBr, the effect of the charge separation was not notable because of an insufficiency of BiOBr. Nevertheless, the introduction of an excessive amount of BiOBr affected the intimate contact between BiOBr nanoplates and ZTO nanoparticles, and the efficient charge carrier separation was hindered correspondingly when increasing BiOBr mass ratio beyond the optimum value (50%). Thus, an appropriate mass ratio of BiOBr is pivotal to modulate the photodegradation behavior of ZTO/BiOBr nanocomposites.

Considering that the photolysis of RhB process approximately obeys a pseudo-first-order kinetic equation of −ln(C/C_0_) = kt, in which C and C_0_ refer to the RhB concentration at time t (min) and t = 0; k (min^−1^) represents the apparent reaction rate constant, the plots of −ln(C/C_0_) with respect to irradiation time are depicted in Figure 6c. The k value for RhB photodegradation over different photocatalysts followed the order of 1:1 > 2:1 > 1:2 > 4:1 > BiOBr > ZTO. Particularly, the ZTO/BiOBr nanocomposites with mass ratio of 1:1 possess the highest k value (0.037 min^−1^), which is almost 2.7 and 2.1 times greater than that of pure ZTO and BiOBr respectively. The above result was consistent with the conclusions drawn from photocatalytic degradation plots. Taking consideration into practical applications, the stability of the 1ZTO/1BiOBr photocatalyst was investigated by cyclic photodegradation experiments under identical conditions. From Figure 6d, we can observe that there was no evident deterioration in the photcatalytic performance after five cycle runs. The photodegradation rate even exceeded 93% after five cycles, suggesting that the 1ZTO/1BiOBr photocatalyst takes on adequate stability and long life for degrading pollutants.

**Figure 7:**
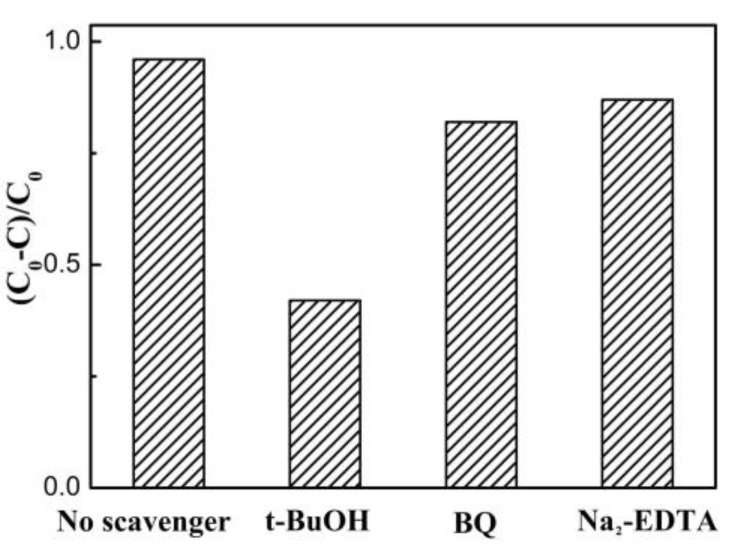
Effect of various scavengers on the visible light photocatalytic performance of 1ZTO/1BiOBr toward the degradation of RhB.

To definitively determine the role of the main active species, such as •OH, •O_2_^−^, and h^+^, in RhB photodegradation process, different scavengers of EDTA disodium (Na_2_-EDTA 4 mmol L^−1^), tert-butyl-alcohol (*t*-BuOH 4 mmol L^−1^), and benzoquinone (BQ 4 mmol L^−1^) were employed as quenchers to capture h^+^, •OH, and •O_2_^−^, respectively. Figure 7 displays the effect of various quenchers on the photodegradation of RhB after 70 min of visible light irradiation over a 1ZTO/1BiOBr photocatalyst. Only a bit loss of photodegradation rate was found for the 1ZTO/1BiOBr photocatalyst after the introduction of BQ or Na_2_-EDTA, whereas the introduction of *t*-BuOH gave rise to a significant decrease of the photodegradation rate, demonstrating that •OH acted as the main oxidative species, and dominated the photodegradation of RhB over the ZTO/BiOBr nanocomposites under visible light irradiation.

**Figure 8:**
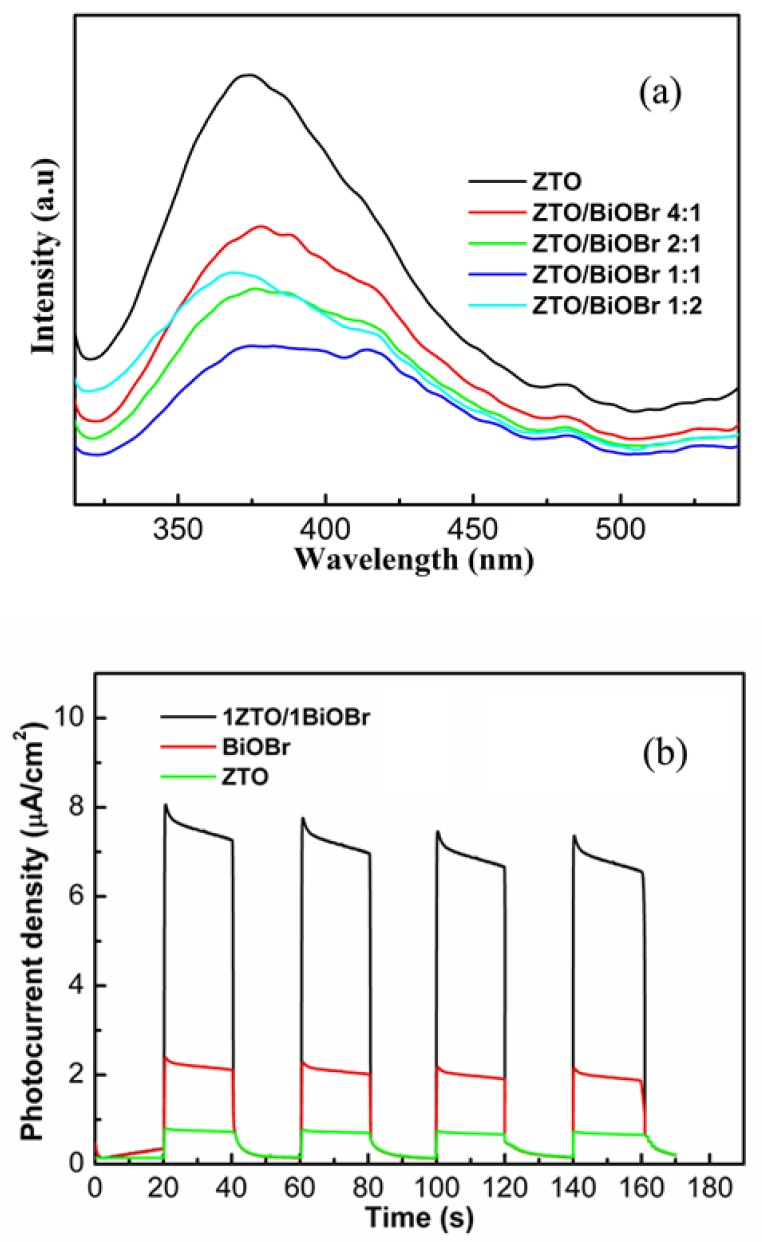
(**a**) PL spectra of pure ZTO, BiOBr, and ZTO/BiOBr nanocomposites; (**b**) Photocurrents of pure ZTO, BiOBr, and 1ZTO/1BiOBr electrodes under visible light irradiation (λ > 420 nm).

As previous studies have documented [30,31], photoluminescence (PL) spectra of semiconductor photocatalysts are intimately related to the migration, transfer, and the recombination rate of photo-induced e_CB_^−^-h_VB_^+^ pairs. Theoretically, the PL emission originates from the recombination of charge carriers, and the intensity of PL emission peaks is approximately proportional to the recombination amount of the excited e_CB_^−^-h_VB_^+^ pairs. In other words, lower intensity of the PL emission peak means that the higher the separation efficiency of photo-induced e_CB_^−^-h_VB_^+^ pairs is available; accordingly, high photodegradation performance will be achieved. Based on the above, PL spectra of the fabricated photocatalysts were measured, and the results are presented in Figure 8a. The strong emission peak located about 370 nm corresponds to the band-band PL phenomenon of the photo-induced charge carriers for ZTO. Two weak emission peaks located at about 420 nm and 480 nm could be attributed to either to oxygen vacancies or to other kinds of defects, e.g., tin vacancies and O_2_ interstitials [32,33]. Among these photocatalysts, pure ZTO showed the strongest PL emission intensity, suggesting a high charge carrier recombination rate. As for ZTO/BiOBr nanocomposites, the PL emission peak intensity decreased continuously, indicating that the recombination of photo-generated charge carriers was effectively restricted due to the efficient charge injection between the two components. The 1ZTO/1BiOBr photocatalyst showed the weakest recombination PL intensity compared to other photocatalysts, revealing the smallest recombination rate of photo-induced e_CB_^−^-h_VB_^+^ pairs in the hybrid system. The increased separation efficiency would provide more opportunities for photogenerated carriers to form active radicals involved into the photodegradation of RhB. The transient photocurrent test was also performed to examine the separation and transfer of photo-excited holes-electrons pairs over different photocatalysts. As displayed in Figure 8b, the photocurrents followed the order of 1ZTO/1BiOBr > BiOBr > ZTO, suggesting that 1ZTO/1BiOBr photocatalyst has a more efficient separation and migration efficiency of the photoexcited carriers. Accordingly, the result of the photocurrent is in good agreement with that of PL spectra.

**Figure 9:**
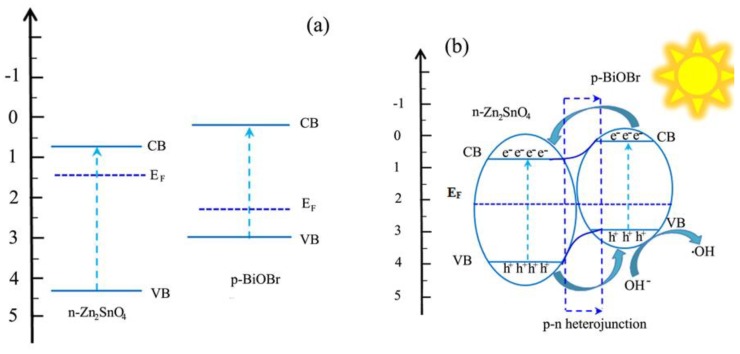
Schematic illustration of the energy band structures (**a**) for separate phases and (**b**) after the formation of *p*-*n* heterojunction of ZTO and BiOBr.

As demonstrated above, the 1ZTO/1BiOBr photocatalyst showed the greatest separation efficiency and highest photocatalytic performance. In order to disclose the enhancement of the photocatalytic performance of ZTO/BiOBr nanocomposites, a possible photocatalytic mechanism is proposed and illustrated in Figure 9. Under visible light illumination, BiOBr can be easily excited to produce electron-hole pairs, owing to its appropriate band gap. For ZTO, a small portion of electrons located at the VB position of a shallow energy level is able to transfer to the CB portion, due to the existence of defects, subsequently, generated electron-hole pairs are also achieved on the surface of ZTO. On the basis of the calculation result, the two components of ZTO (*n*-type) and BiOBr (*p*-type) have well-matched energy band structures. After contact, *p*-*n* junctions are formed along the interface of the two components. The arrangement of VB and CB energy band levels induced by the potentials’ difference leads to the formation of an inner electric field with the direction from *n*-type ZTO to *p*-type BiOBr, promoting different Femi levels to reach an equilibrium between the two components. As depicted in Figure 9, the CB position of BiOBr is more negative than that of ZTO, so the photogenerated electrons in the CB of BiOBr are capable of transferring to the CB of ZTO, based on the charge separation way. Simultaneously, the VB position of ZTO is more positive than that of BiOBr; thus, some available holes migrate from the surface of VB of ZTO to that of VB of BiOBr. Based on the above analysis, photogenerated electron-hole pairs are effectively separated by assisting the functionality of the inner electric field. Furthermore, the VB potential of of BiOBr (3.07 eV vs. NHE) is more positive than that of OH^−^/•OH (1.99 eV) [34,35]; thus, more photogenerated holes can react with OH^−^ to form •OH, affecting the oxidation reaction of the RhB molecules. This result is highly consistent with the conclusion of the trapping experiment, i.e., that OH played a dominant role in the oxidation reaction process.

## 3. Experimental Section

### 3.1. Materials

All chemicals, including tin (IV) chloride penthydrate (SnCl_4_·5H_2_O), zinc acetate dehydrate (Zn(CH_3_COO)_2_·2H_2_O), sodium hydroxide (NaOH), bismuth nitrate pentahydrate (Bi(NO_3_)_3_·5H_2_O), potassium bromide (BiOBr), and rhodamine B (RhB) were purchased from Sinopharm Chemical Reagent CO., Ltd., Shanghai, China, and used as received.

### 3.2. Synthesis Zn_2_SnO_4_ Nanoparticles (ZTO NPs)

ZTO NPs were prepared via a simple hydrothermal synthesis route; the detailed procedure was already described in our previous studies [8]. Briefly, after the hydrothermal treatment, a white precipitate was collected, centrifuged and washed repeatedly with deioned water and ethanol respectively, followed by drying at 60 °C for 12 h.

### 3.3. Synthesis ZTO/BiOBr Nanocomposites

Typically, ZTO/BiOBr nanocomposites were synthesized via a facile in situ precipitation method. 2 mmol of KBr was dissolved into 60 mL deionized water under vigorous stirring and formed a transparent solution; then a certain amount of ZTO NPs was completely dispersed into solution containing KBr under vigorous magnetic stirring, followed by ultrasonic treatment for 30 min. After that, 2 mmol Bi(NO_3_)_3_·5H_2_O dissolved into 40 mL ethylene glycol was added dropwise into the previous suspension under constant stirring. Subsequently, the mixture was continuously stirred for another 3 h at 40 °C to make the precipitation reaction complete. After centrifuging and washing with deioned water and ethanol repeatedly, the resulting product was dried at 60 °C in an oven overnight. By varying amount of ZTO NPs, a series of ZTO/BiOBr nanocomposites with mass ratios of 4:1, 2:1, and 1:1, 1:2 (denoted as 4ZTO/1BiOBr, 2ZTO/1BiOBr, 1ZTO/1BiOBr and1ZTO/2BiOBr) was obtained via the similar synthetic process. For comparison, bare BiOBr was also prepared by the similar procedure described above without the addition of ZTO NPs.

### 3.4. Characterization

Morphological observation and microstructural analysis was taken on a JEM 2100F transmission electron microscope (JEOL Ltd., Tokyo, Japan) and a Hitachi S-4800 field emission scanning electron microscope (Hitachi, Tokyo, Japan). The crystalline structure identification was accomplished on a D8 Advance X-ray diffractometer (Bruker, Billerica, MA, USA) with a Cu Kα source (λ = 0.15406 nm). The XRD measurement was performed at 40 kV and 40 mA in the range of 10°–80°. The surface composition was analyzed by X-ray photoelectron spectroscopy (XPS) spectra on an ESCALAB 250Xi spectrometer (Thermo Fisher Scientific, NewYork, NY, USA) with a monochromatic Al-Kα radiation source. UV-vis diffuse reflectance spectra and room temperature photoluminescence (PL) spectra were recorded on a TU 1901 UV-vis spectrophotometer (Puxi, Beijing, China) with BaSO_4_ as reference and a LS55 fluorescence spectrophotometer (PE, Waltham, MA, USA) with an excitation wavelength of 310 nm, respectively. The Brunauer-Emmett-Teller (BET) specific surface area of the as-prepared pure ZTO and 1ZTO/BiOBr samples was measured on a Quantachrome NOVA 2000e sorption analyzer.

### 3.5. Photocatalytic Experiments

The process of the photodegradation test was demonstrated in our previous studies [16,29,36,37]. In short, two daylight lamps (60 W, λ ≥ 420 nm) were designated as the visible light source for triggering the photodegration reaction of RhB. In a typical run, a thin layer of film (100 mg photocatalyst) was dispersed into 40 mL RhB aqueous solution (1.0 × 10^−5^ M). After a certain interval of visible light illumination, the reacted solution treated by centrifuging was analyzed by using an UV-vis spectrophotometer at its characteristic absorbance wavelength of 554 nm. The C/C_0_ ratios of the RhB concentrations were employed to evaluate the degradation efficiency, in which C represented the concentration at a certain irradiation time, while C_0_ was the initial concentration after adsorption-desorption equilibrium.

### 3.6. Photocatalytic Measurements

Transient photocurrents of the prepared photocatalysts were performed on an electrochemical workstation (CHI660E, Chenhua Instruments Co., Shanghai, China) using a standard three electrode system with a Pt wire as the counter electrode, a saturated Ag/AgCl electrode as the reference electrode, and the as-prepared photocatalysts as the working electrode, respectively. The detailed procedure of the measurement was reported in our previous study [38].

## 4. Conclusions

In summary, ZTO/BiOBr hybrid nanocomposites have been successfully constructed via a facile approach. The introduction of BiOBr resulted in the establishment of the contact interface of Zn_2_SnO_4_/BiOBr heterojunctions that endowed the photocatalyst with an improved light harvesting capacity, high efficiency of photogenerated charge separation, as well as stronger oxidation ability. Zn_2_SnO_4_/BiOBr hybrid photocatalysts displayed enhanced photocatalytic performance and excellent anti-photocorrosion toward the photodegradation of RhB under visible light illumination. This study will open up a new way of constructing novel hybrid photocatalysts with efficient visible-light phtocatalytic activity for use in the fields of environmental remediation and hydrogen energy production.
